# Laboratory evaluation of sugar alcohols for control of mosquitoes and other medically important flies

**DOI:** 10.1038/s41598-022-15825-z

**Published:** 2022-08-12

**Authors:** Ilia Rochlin, Gregory White, Nadja Reissen, Dustin Swanson, Lee Cohnstaedt, Madeleine Chura, Kristen Healy, Ary Faraji

**Affiliations:** 1 Center for Vector Biology, Rutgers University, 180 Jones Avenue, New Brunswick, NJ 08901 USA; 2 Salt Lake City Mosquito Abatement District, 2215 North 2200 West, Salt Lake City, UT 84116 USA; 3 USDA-ARS, Arthropod Borne Animal Disease Research Unit, 1515 College Avenue, Manhattan, KS 66502 USA; 4 USDA-ARS, Foreign Arthropod Borne Animal Diseases Research Unit, 1515 College Avenue, Manhattan, KS 66502 USA; 5 Entomology Department, Louisiana State University, 404 Life Sciences, Baton Rouge, LA 70803 USA

**Keywords:** Entomology, Malaria

## Abstract

Insecticide application for vector control is the most controversial component of a public health program due to concerns about environmental and human health safety. One approach to overcome this challenge is the use of environmentally benign active ingredients. Among the most promising emerging strategies are attractive toxic sugar baits. Sugar alcohols—naturally occurring molecules safe for human consumption but potentially toxic to insects when ingested, have received increased attention for use with this approach. For this study, we screened the toxicity of four different sugar alcohols on several mosquito species, a biting midge, and a filth fly. Sugar alcohol mortalities exceeded those in the sucrose (positive control) only group. However, only erythritol and highly concentrated xylitol induced mortalities exceeding those in the water only (negative control) treatment ranging from approximately 40–75%. Formulations containing erythritol and xylitol should be further investigated under field conditions for efficacy in reducing populations of biting flies and for assessing potential non-target impacts.

## Introduction

Effective mosquito control relies on a multi-faceted approach known as integrated mosquito management to reduce biting pressure and to decrease the transmission of mosquito-borne pathogens^1^. Integrated mosquito management utilizes a variety of methods, including surveillance of mosquitoes and the pathogens they transmit, and an assortment of physical, biological, and chemical control techniques. Ultimately, the use of insecticides represents a large component of the control efforts^2,3^. As part of an integrated mosquito management approach, multiple life stages of mosquitoes are targeted with pesticides using different active ingredients.

For over half a century, adult mosquito control has relied primarily on topical insecticides. These products are either sprayed as fine droplets into the air in ultra-low volume (ULV) or thermal fog applications, or applied as residual barrier treatments to surfaces where mosquitoes are likely to land. When applied indoors, these are commonly known as indoor residual sprays (IRS)^4^. Residual treatments can also be applied to bed nets in malaria endemic areas^5^. These methods have been successful, but they have also led to insecticide resistance in many places^3,6–8^.

In the US, publicly funded adult mosquito control relies almost solely on area-wide ULV applications, whereas the private pest control industry applies mostly residual pesticide treatments. Only two classes of active ingredients are routinely used by both public agencies and privates companies—organophosphates and pyrethroids^2,9^. Lack of additional active ingredients and sparsity of mosquito control products compared to the agricultural analogs have led to the rise of insecticide resistance^10–12^. The heavy use of these same active ingredients by pesticide applicators in agriculture, landscape, and structural pest control is another probable contributing factor to resistance^13,14^.

Alternative insecticides for mosquito control are needed for two main reasons: safety and insecticide resistance. Insecticide use is typically the most controversial component of a program due to concerns about environmental and personal safety. The main approaches to overcome this challenge have been to limit insecticide applications geographically and temporarily, and only conduct applications when deemed necessary, as determined by surveillance. Product rotation and alternative pesticides with more environmentally benign active ingredients can be used to combat insecticide resistance and limit ecological impacts. The World Health Organization has publicly recognized these challenges and has been promoting the development of new pesticide products to reduce environmental impacts and to prevent insecticide resistance^15^.

One of the newer emerging novel control strategies are attractive toxic sugar baits (ATSB) for adult mosquito control^16–18^. The use of toxic baits to kill insects has been used for decades to reduce populations of many pests such as ants, cockroaches, and termites^19,20^. With the exception of pioneering studies in the 1960s using malathion^21^, this approach has not been employed for mosquito control until the last decade^16^. Adult mosquitoes require sugar as an energy source^22,23^, and this requirement is used as the physiological basis for this method of insecticide delivery. Trials with ATSB have demonstrated great success in reducing mosquitoes in a number of different laboratory, semi-field, and field trials^16,17,24^. Numerous active ingredients have been evaluated in conjunction with ATSB, from conventional pesticides and mosquito adulticides to ingredients exempt from the U.S. Environmental Protection Agency (EPA) pesticide regulations^25^. These exempt products, mostly different botanical oils such as garlic oil^26^, offer a major advantage over more traditional pesticides because they are not required to undergo the customary regulatory process to acquire EPA approval, which can be long and expensive. Thus, an exempt chemical that is stable, non-repellent, easy to formulate, and has been determined to be safe for people and the environment while also exhibiting toxicity to mosquitoes would be an ideal candidate for ATSB methods.

Recently, the insecticidal properties of sugar alcohols, also known as polyols^27,28^ have received increased attention^29^. Sugar alcohols are naturally occurring molecules found in many plants, and have many qualities that make them ideal candidates for ATSBs. A number of various sugar alcohols such as erythritol, sorbitol, xylitol, and mannitol are considered safe for human consumption by the U.S. Food and Drug Administration (FDA) and the European Union, and they are already commonly used as reduced calorie sweeteners^28,30,31^. Erythritol has shown promising insecticidal activity on fruit flies, fire ants, and the yellow fever mosquito, *Aedes aegypti* L.^27,29,32–36^. The objectives of this study were to (1) test various sugar alcohols for insecticidal activity against biting flies (Diptera) of public health importance and (2) determine if susceptibility of sugar alcohols varied across these different species.

## Results

**Figure 1:**
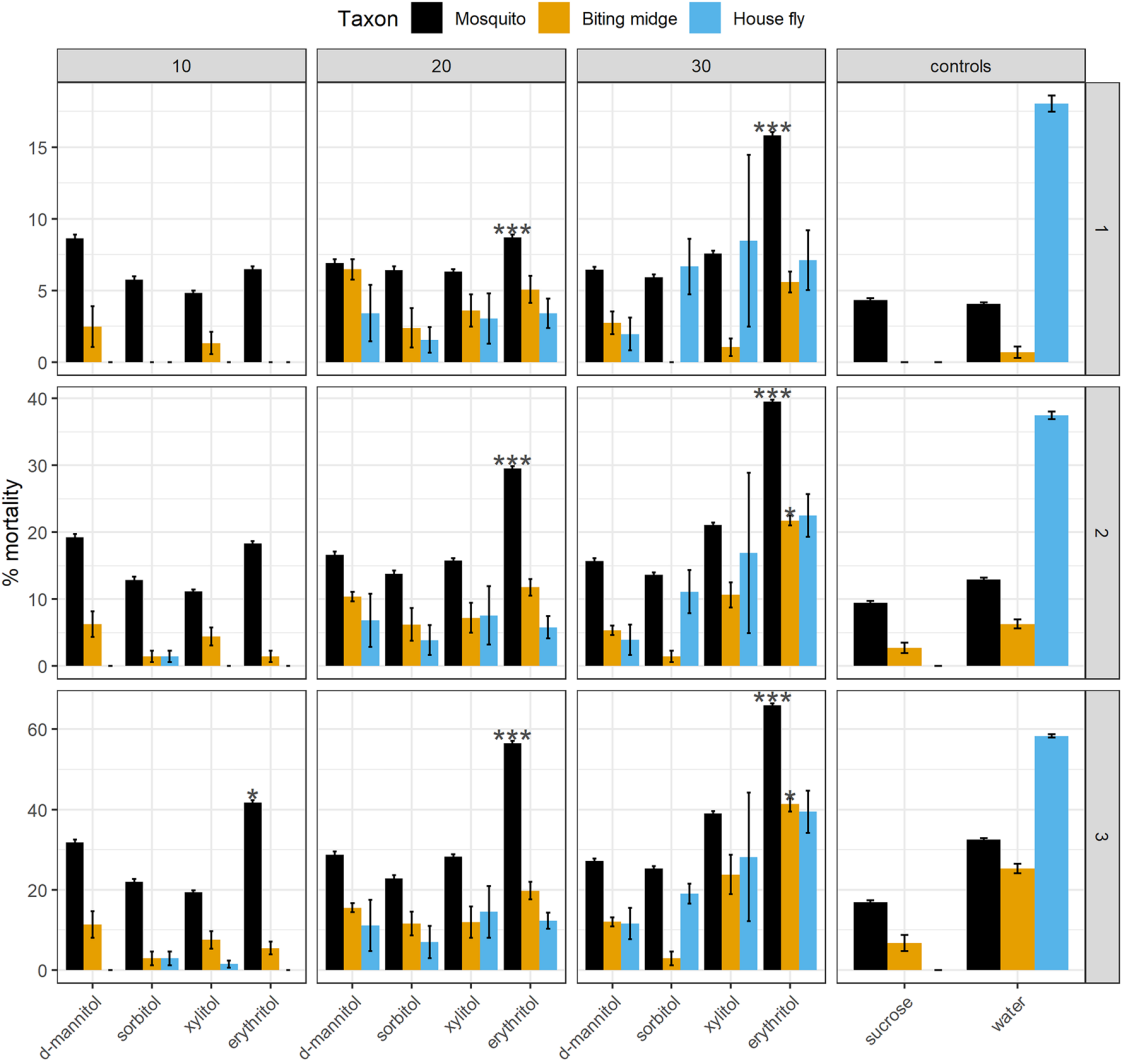
Cumulative percent mortality. Barplots show average mortality with associated standard error bars. Concentrations (10%, 20%, and 30%) are in columns and days 1 through 3 are in rows (not all experiments continued through day 4). Controls, i.e.10% sucrose and distilled water are shown in the right panel. Diptera included the following species (sample size indicated in parenthesis): Mosquitoes (Mosq) *Ae. aegypti* (n = 727), *Ae. dorsalis* (n = 3283), *Cx. quinquefasciatus* (n = 1868), and *Cx. tarsalis* (n = 2495); biting midges (CU) *Cu. sonorensis* (n = 836); house or filth flies(MU) *Mu. domestica* (n = 820). Asterisks indicated statistical significance for those treatment groups where mortality exceeded those of water only control (starvation) at *P < 0.05, **P < 0.01, ***P < 0.001.

Mortality rates were compared to a sucrose only fed group (expected high survival, positive control) and to a water only, i.e. starvation group (expected low survival, negative control). Mortalities in the sucrose only group ranged from none in the common housefly, *Musca domestica*, to 4–7% in colonized mosquito and biting midge species (*Aedes aegypti, Culex quinquefasciatus*, and *Culicoides sonorensis*) to 21–29% in wild caught mosquitoes (*Ae. dorsalis* and *Cx. tarsalis*) on day 3 of the study (Supplementary Table 1). Among all sugar alcohol concentrations, only erythritol at 20% and 30% concentrations consistently exceeded the mortality rates for water only (Fig. 1, Supplementary Table 1). All four mosquito species (*Ae. aegypti*, *Ae. dorsalis*, *Cx. quinquefasciatus*, and *Cx. tarsalis*) had significantly higher mortalities compared to water at 20% and 30% erythritol concentrations (Tukey’s range test adjusted pairwise comparisons *P* < 0.05). On day 3, 30% erythritol mortalities compared to water only groups (mean% ± SE) were, respectively: 73.0 ± 0.5 vs. 18.6 ± 6.3 for *Ae. aegypti*, 71.1 ± 0.4 vs. 44.4 ± 5.5 for *Ae. dorsalis,* 58.8 ± 7.1 vs. 26.8 ± 3.2 for *Cx. quinquefasciatus*, and 63.9 ± 4.1 vs. 32.5 ± 5.1 for *Cx. tarsalis*.

Among non-mosquito Diptera, *Cu. sonorensis* had significantly higher mortality compared to the water only group at 30% (but not at 20%) erythritol (Tukey’s range test adjusted pairwise comparisons *P* = 0.003). On day 3, *Cu. sonorensis* mortalities were 41.3 ± 3.2 (30% erythritol) and 25.3 ± 2.1 (water only), whereas sucrose only mortality was at 6.7%. *Musca domestica* was the only species in which 30% erythritol mortality was significantly lower than that of the water only group (*P* < 0.001). On day 3 of the experiment *Mu. domestica* mortalities were 39.4 ± 9.1 (30% erythritol) and 58.3 ± 0.7 (water only), whereas no mortality occurred in the sucrose only group. Apart from erythritol, xylitol fed groups at the highest sugar alcohol concentration of 30% exhibited elevated mortalities (Fig. 1, Supplementary Table 1). However, the only significant difference between 30% xylitol and the water only group over the course of the study was observed for *Cx. quinquefasciatus* (*P* < 0.05).

**Figure 2:**
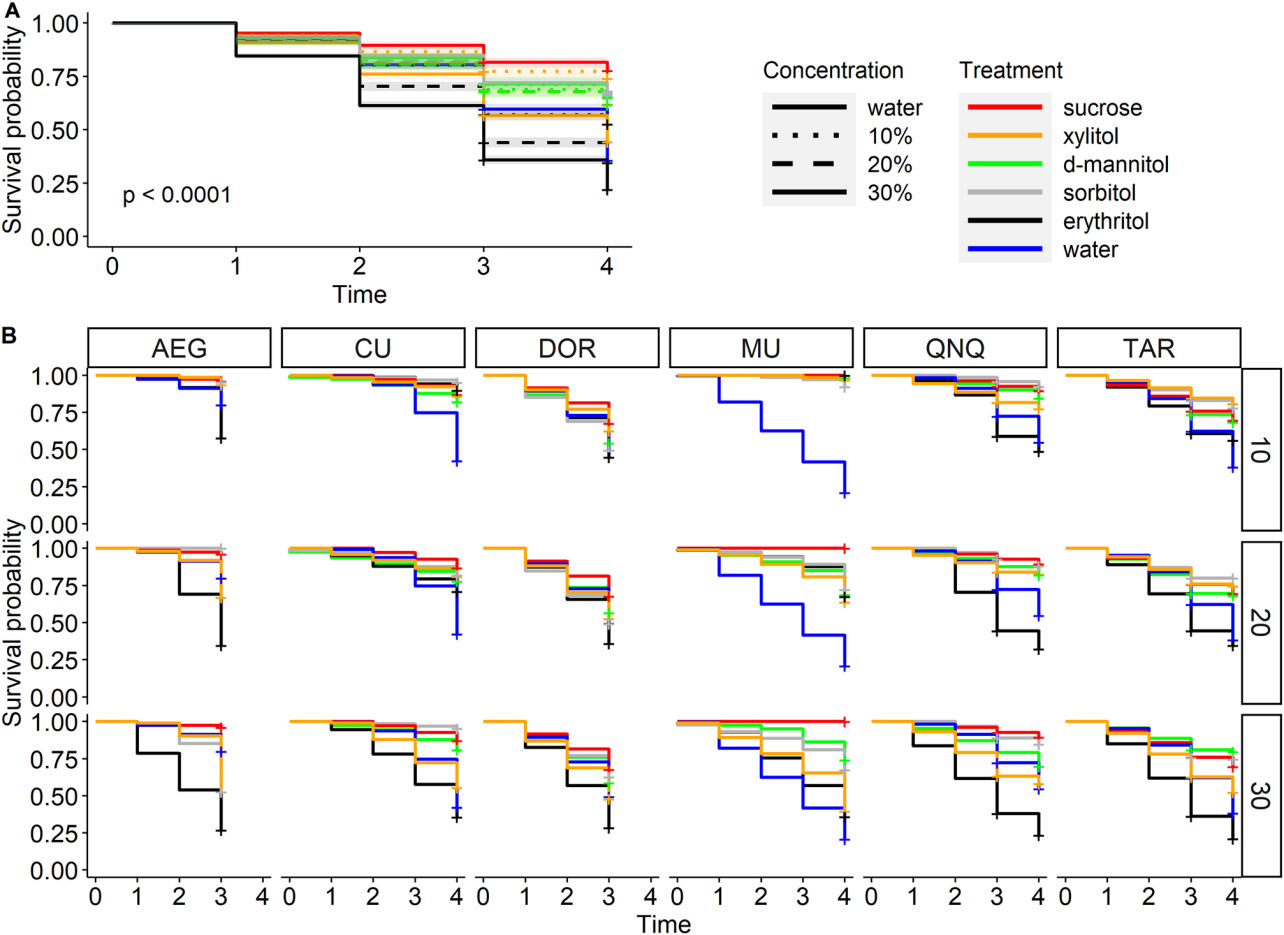
Kaplan–Meier survival curves. (**A**) Survival curves combined for all species. Concentration is indicated by the line type, and different treatments by color. The 95% confidence intervals are in light grey. (**B**) Survival curves faceted by species (columns) and sugar alcohol concentration (rows) over time from day 0 through day 4. Different colors indicate different sugar alcohols or treatments (see panel (**A**) for treatment color coding). AEG—*Ae. aegypti*, CU—*Cu. sonorensis*, DOR—*Ae. dorsalis*, MU—*Mu. domestica*, QNQ—*Cx. quinquefasciatus*, TAR—*Cx. tarsalis*.

The overall survival rates for all Diptera species combined were determined via a Kaplan–Meier estimator (Fig. 2A). The median survival time was estimated at 3 days for 20% and 30% erythritol, and 4 days for water and 30% xylitol. The median survival time exceeded the maximum duration of the experiments (4 days) for all other treatments. The survival times for the 20% and 30% erythritol treatment groups were significantly lower than the survival times for water, 30% xylitol, and 10% erythritol groups (*P* < 0.001). The survival time for the 30% erythritol treatment group was significantly lower than that for the 20% erythritol treatment group (*P* < 0.001). Survival times for 30% xylitol, and 10% erythritol groups were similar to that of water only treatment (*P* = 0.880 and *P* = 0.055, respectively). The survival times for all other treatments were significantly higher than that of the water only group (*P* > 0.05).

The survival rates for individual species followed similar pattern with some variation in *Cu. sonorensis* and *Mu. domestica* (Fig. 2B). The lowest median survival times of 3 days for all four mosquito species were determined for the 20% and 30% erythritol treatment groups. The survival times were significantly lower compared to all other treatments including the water only group (all pairwise *P* < 0.001). The survival times for 30% erythritol treatment groups was invariably significantly lower compared to that of the 20% erythritol treatment group for all four mosquito species (all pairwise *P* < 0.002). The survival times for 10% erythritol and water only treatment groups were lower compared to other treatments for *Cx. quinquefasciatus*, and *Cx. tarsalis* (all pairwise *P* < 0.05). However, *Ae. aegypti* survived longer on water only compared to 10% erythritol as well as 30% sorbitol and 30% xylitol (*P* < 0.0045); the survival times for the three sugar alcohol treatments were similar to each other (*P* > 0.05). For *Ae. dorsalis*, most treatments (except 10% xylitol, 20% and 30% d-mannitol, and 30% sorbitol that had higher survival times) appeared to have similar survival times to those for water and 10% erythritol groups (*P* < 0.05).

For *Cu. sonorensis*, the lowest median survival times was 4 days for 30% erythritol and water treatment groups. The survival time for 30% erythritol group was significantly lower than that of water only group (*P* = 0.03919) and significantly lower compared to all other treatments (*P* < 0.001). The survival time for water only group was similar to that of 30% xylitol (*P* = 0.2239) and significantly lower compared to all other treatments (*P* < 0.01). In turn, the survival time for 30% xylitol group was similar to that of 20% erythritol (*P* = 0.0899) and significantly lower compared to all other treatments (*P* < 0.01). *Musca domestica* response to treatments differed even more from those of the mosquito species. Water only group had the lowest median survival time of 3 days (all pairwise *P* < 0.001) followed by 30% erythritol and 30% xylitol treatment groups that had the median survival times of 4 days similar to each other (*P* = 0.5036). These two treatments had significantly lower survival times compared to all other treatments (all pairwise *P* < 0.001).

**Figure 3:**
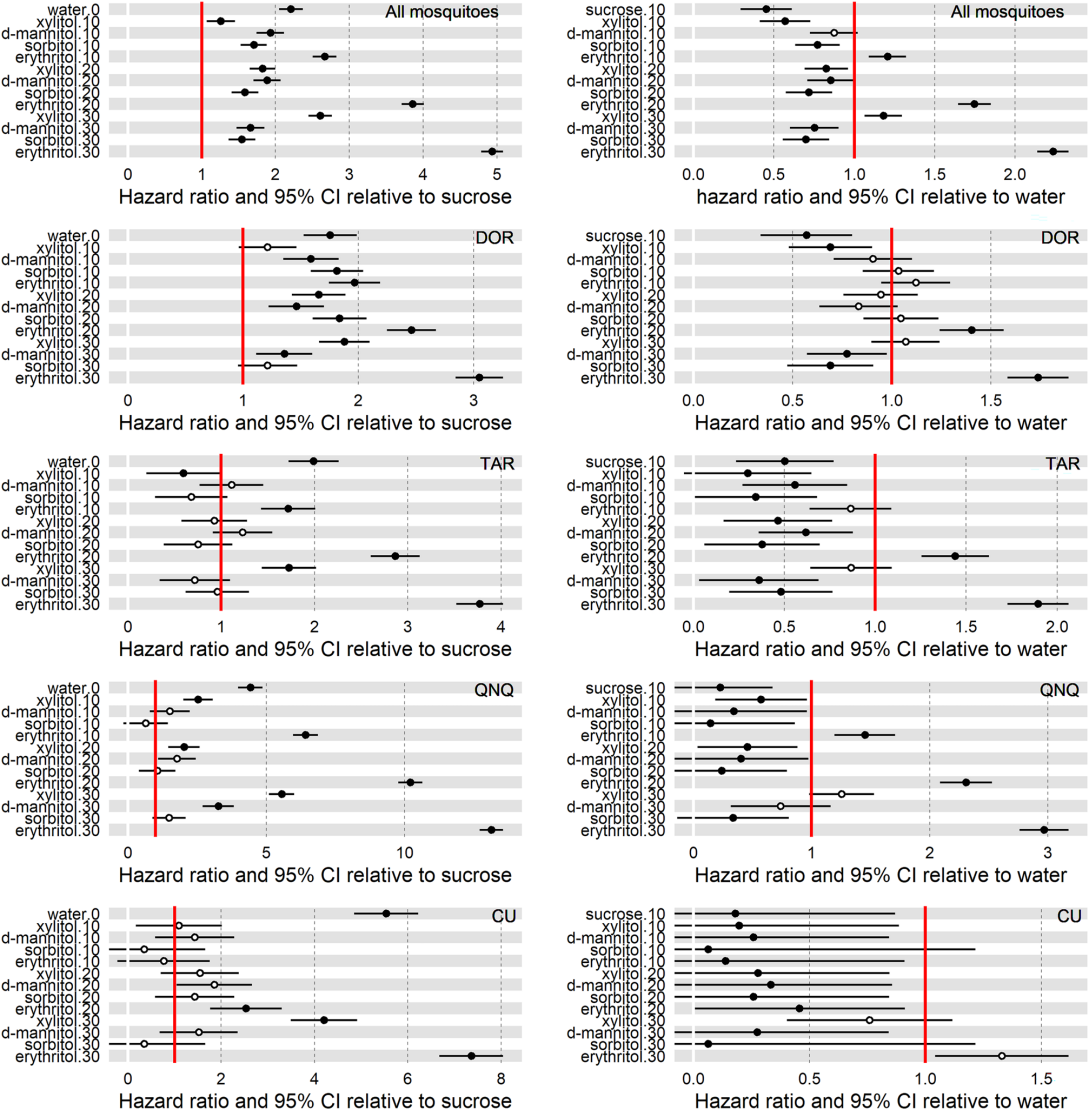
Hazard ratios for different treatments. Hazard ratio displays the mortality rate in the treatment group compared to that in the reference group: i.e. hazard ratio of 2.0 designates that flies are dying at twice the rate compared to the reference. Hazard ratio of 1.0 (red bar on the plots) designates no difference. Sugar alcohol concentrations are indicated by numbers after the treatment’s name. Rows: combined mosquito species (top row), separate species in each following row: DOR—*Ae. dorsalis*, TAR—*Cx. tarsalis,* QNQ—*Cx. quinquefasciatus*, CU—*Cu. sonorensis*. Data from *Ae. aegypti* and *Mu. domestica* experiments violated Cox regression assumptions and were omitted from the individual species analysis (*Ae. aegypti* was included among combined mosquito species). Left column: Hazard ratio relative to 10% sucrose (positive control) Right column: Hazard ratio relative to water (i.e. starvation, negative control). The 95% confidence intervals and statistical significance at *P* < 0.05 (filled circles) are shown. Open circles indicate non-significant differences at *P* > 0.05.

Hazard ratios (HR) calculated from Cox regression represent the increase in mortality in the treatment groups compared to the reference—either sucrose (positive control) or water (negative control) only groups (Fig. 3).

For mosquitoes, the mortality rates were significantly elevated in all treatment groups compared to those insects fed on 10% sucrose solution (Fig. 3, all *P* < 0.001). However, HR > 2.0 (i.e., more than twice as likely to die compare to the sucrose fed insects) was observed only for those mosquitoes exposed to erythritol (all concentrations), 30% xylitol, and water only. Two erythritol treatments had the highest HRs: 30% erythritol HR = 4.9 [4.3–5.7] and 20% erythritol HR = 3.9 [3.3–4.5] followed by 10% erythritol HR = 2.7 [2.3–3.1], 30% xylitol HR = 2.6 [2.2–3.1], and water only HR = 2.2 [1.9–2.6] treatments. Compared to the water only group for all mosquito species, 10% erythritol HR = 1.2 [1.1–1.4], 20% erythritol HR = 1.3 [1.2–1.5], 30% erythritol HR = 1.8 [1.6–1.9], and 30% xylitol HR = 1.2 [1.1–1.3] were the only treatments with significantly higher HRs (Fig. 3, *P* < 0.006).

Comparing Cox hazard ratios for individual mosquito species (Fig. 3) revealed that 20% and 30% erythritol HRs consistently exceeded that of the water only treatment. In contrast, for the biting midge *Cu. sonorensis*, 30% xylitol had HR = 4.2 [2.1–8.6] comparable to 30% erythritol HR = 7.4 [3.7–14.5] and water only HR = 5.5 [2.8–11.0] relative to sucrose fed group. Both 30% xylitol and 30% erythritol HRs were similar to that of water only (*P* > 0.05), whereas all other treatments had significantly lower HRs, all *P* < 0.001 (Fig. 3). Individual Cox hazard ratios were not calculated for *Ae. aegypti* and *Mu. domestica* because the proportional hazards assumption was not supported by the data.

## Discussion

The main objective of this study was to evaluate and compare several common sugar alcohols for their insecticidal activity against mosquitoes, biting midges, and house flies. All four sugar alcohols used in this study are approved for human consumption in the US and Europe^28,31^ and are exempt from the requirements for conventional pesticide registration^37^. Erythritol, sorbitol, xylitol, and d-mannitol all have a similar chemical structure, with differences mainly in the length of the carbon chain (reviewed in^28^). Whereas erythritol has a chain composed of four carbon atoms, xylitol has five carbon atoms, and d-mannitol and sorbitol have six carbon atoms each. These relatively minor differences in chemical structure have a significant effect on how these molecules are processed when ingested. In mammals, erythritol does not appear to be metabolized but, is readily absorbed and then excreted in urine^30^. Sorbitol, xylitol, and d-mannitol can be metabolized to some degree by mammals and can be digested by bacteria in the intestines^28^. However, sugar alcohol metabolism in insects is less understood^34^. Erythritol-fed mosquitoes consistently had the lowest levels of trehalose, glycogen, and lipids compared to other sweeteners; thus, it has been hypothesized that erythritol inhibits sucrose metabolism and causes the mosquitoes to starve to death^36^.

Unlike other commonly applied exempt products with insecticidal properties such as essential oils, which are hydrophobic, volatile, and can be repellent to insects^38^, sugar alcohols easily dissolve in water, are stable in solution, and are extensively used for baking and consumption due to high heat stability^28^. Food mixtures containing sucrose and sugar alcohols were readily fed upon by Diptera in trials^27,32,36,39^. Thus, the favorable profile of sugar alcohols as a potential toxicant in ATSBs was established by previous studies^27,32–36,39–41^. These studies utilized model organisms such as fire ants (*Solenopsis invicta* Buren), house flies (*Mu. domestica*), fruit flies [*Drosophila* spp., *Bactrocera dorsalis* (Hendel)], and yellow fever mosquitoes (*Ae. aegypti*). Comparisons of the effects of different sugar alcohols on these insects were generally in line with our study. Erythritol fed insects exhibited the highest mortality followed by xylitol and/or d- mannitol, with sorbitol displaying the lowest mortality among all treatments, as demonstrated by the experiments using house flies (*Mu. domestica*)^39,42^. Sorbitol having no effect on fly mortality is not surprising, as it has also been shown to be readily metabolized by mosquitoes^43^.

*Drosophila suzukii* Matsumura, a fruit fly, fed on erythritol and sucrose mixture had 100% mortality after 6 days under different concentrations, whereas xylitol caused increased mortality only when combined with the lowest sucrose concentration^33^. Similarly, erythritol caused 100% mortality in *Drosophila melanogaster* Meigen with xylitol and d-mannitol groups indistinguishable from the sucrose control at 6 days; however, female flies experienced higher mortality in the d-mannitol group after 17 day exposure^32^. The yellow fever mosquito (*Ae. aegypti*) experienced 100% mortality when fed 10% erythritol after 10 days, which was significantly higher compared to water only as well as sucralose, aspartame, and saccharine treatments^36^. These comparative results were similar to our observations. Diptera fed on erythritol displayed the highest mortalities among all sugar alcohols tested across all concentrations (Fig. 1). Mosquito mortalities in 30% erythritol treatment ranged from approximately 36–46% at 2 days to 59–73% at 3 days. *Culicoides sonorensis* and *Mu. domestica* had lower mortalities ranging from approximately 20% at 2 days to 40% at 3 days. These survivorship trajectories were similar to those observed in the longer duration studies suggesting that 100% mortality would have been reached within the same 7–10 day timeframe.

The effect of erythritol was perhaps expressed more clearly by the hazard ratio differences (Fig. 3). For combined mosquito species, the individual mosquito female was 4–5 times as likely to die in the 20% or 30% erythritol group, respectively, compared to sucrose fed females. The effects of the xylitol were detected only at the highest 30% concentration being comparable to 10% erythritol at the hazard ration of about 2.5 times that of the control sucrose group. For non-mosquito Diptera species, *Cu. sonorensis* and, especially, *Mu. domestica*, the mortality induced by 30% xylitol appeared to be similar to 20% erythritol. In *Mu. domestica*, xylitol was almost as effective as erythritol in the short term^42^ most likely due to the reduced efficacy of the latter in this species^39^. However, erythritol mortality appeared to be higher over the longer time period of about 2 weeks^42^.

Since erythritol was identified as the most potent and promising insecticide among sugar alcohols in several investigations^32,33,35^, several previous studies focused on characterizing its effects on Diptera^27,34,36,41^. Erythritol toxicity was dose dependent in *D. melanogaster*^27^, *D. suzukii*^33,34^, and *Ae. aegypti*^41^. Similarly, in our study, erythritol response was dose dependent with significantly higher mortality and associated hazard ratios, and lower survivorship at higher concentrations for all mosquito species and *Cu. sonorensis*. In the house fly (*Mu. domestica*), the water only group mortality was at least equal to and exceeded that of erythritol treatment suggestion that starvation rather than insecticidal activity were the primarily mode of action^39^. Based on our data, starvation could not be ruled out for *Cu. sonorensis* because the water only mortality was only surpassed by the highest concentration of erythritol. However, this clearly was not the case for the four mosquito species, for which water only treatments had significantly lower mortalities and higher survivorship compared to erythritol indicating toxic effects of this sugar alcohol rather than starvation effect. Ingestion of erythritol inhibits sucrose metabolism^36^. Specifically, erythritol was an inhibitor of α-glucosidase, an enzyme that breaks down starch and disaccharides to glucose, in the true bug *Dysdercus peruvianus* Guerin-Meneville^44^. There may be additional toxic effects of erythritol in insects. In *Ae. aegypti* mosquitoes, mannose-1-phosphateguanyltransferase gene was significantly upregulated in erythritol fed females compared to water and sucrose implicating protein glycosylation in the toxicity of erythritol^36^. Erythritol also contributed to an increased osmotic pressure in the hemolymph of *D. suzukii*, as a large amount of erythritol was detected which was approximately 17 times that of sucrose^34^.

Based on our study and previous investigations, different concentrations of erythritol and the highest concentration of xylitol should be further tested under semi-field or field conditions to determine impacts on biting Dipteran populations. Another topic is to determine if combining erythritol or xylitol can work synergistically with other ATSB formulas. Such combinations with different sugars such as arabinose, cellobiose, or lactose were toxic to *Ae. aegypti*^45^ and could make a more effective ATSB solution for mosquito control. Other effective ATSB formulation such as boric acid have also been extensively evaluated under laboratory and field conditions^18^. One concern is that, compared to boric acid, the action of sugar alcohols is relatively slow over a period of many days, which might be more suitable for species with relatively confined ranges such as ants or fruit flies, but less so for more widely dispersed insects such as mosquitoes. However, the effects of sugar alcohols on mosquitoes and other biting Diptera may not be limited to only direct mortality^29^. Non-lethal effects, such as reduced longevity, interrupted motor coordination, or reduced fecundity have also been observed^27,32^. Non-lethal effects may facilitate larger negative impacts on target populations under natural conditions. Other areas of research such as the effects of sugar alcohols on the insect gut microbiome^39^ or on vector competence for various pathogens remain unexplored. Additionally, reliance on a single active ingredient even as effective as boric acid may lead to increased resistance or tolerance. Such tolerance has been demonstrated for the German cockroach (*Blattella germanica* L.)^46^ and can be avoided or minimized by rotating active ingredients with different modes of action such as boric acid and sugar alcohols, for example.

If sugar alcohols are incorporated with ATSBs for operational mosquito control^41^, they will also need to be evaluated for potential impacts on non-target insects under natural conditions. For example, erythritol is known to impact representatives of at least four insect orders (reviewed in^29^). Efficacious and safe mosquito and vector control must not rely solely on a single approach, but should rather take advantage of a unified integrated procedure using new methods, equipment, and formulations. Sugar alcohols could potentially enhance such a novel approach to improve quality of life, while protecting public health and reducing chemical insecticide usage for increased environmental sustainability.

## Methods

### Diptera collection, rearing, and maintenance

The experiments were conducted at three locations: Salt Lake City Mosquito Abatement District in UT (SLCMAD), Louisiana State University in Baton Rouge, LA (LSU), and US Department of Agriculture, Manhattan, KS (USDA), see Table 1 for summary. For SLCMAD experiments, *Aedes dorsalis* Meigen and *Culex tarsalis* Coquillett mosquitoes were collected using CO_2_-baited Clarke ABC traps (Clarke, St. Charles, IL, USA) with modified collection boxes. The standard collection net was replaced with a 24 L plastic tote (Sterilite Clear View Latch, Townsend, MA, USA) connected to the trap with a stockinette sleeve. Two 25 × 38 cm rectangular opening were cut into the sides and covered with mesh to allow airflow. A 50 ml conical tube was attached to the inside of the container with hook and loop tape, so the opening was facing upwards. A piece of sponge that was slightly taller than the tube was placed into the opening and then the tube was filled with 10% sucrose. Collection containers were brought back the laboratory where mosquitoes were aspirated and sorted. Only female mosquitoes were used in assays.

**Table 1:** Experimental setup overview: Diptera species used, test locations, number of trials, and duration.

Species	Source	Sex	Test Location	Sugar alcohols	# trials	Duration (days)
*Ae. dorsalis*	Wild	F	SLCMAD	All (4)	3	3
*Cx. tarsalis*	Wild	F	SLCMAD	All (4)	3	3
*Cx. quinquefasciatus*	Colony	F	LSU	No d-mannitol (3)	1	3
*Ae. aegypti*	Colony	F	LSU	No d-mannitol (3)	1	3
*Cx. tarsalis*	Colony	F	USDA	All (4)	1	4
*Cx. quinquefasciatus*	Colony	F	USDA	All (4)	1	4
*Cu. sonorensis*	Colony	F	USDA	All (4)	1	4
*Mu. domestica*	Colony	Both	USDA	All (4)	1	4

For LSU laboratory assays, *Aedes aegypti* L.*,* Rockefeller strain, mosquitoes and *Culex quinquefasciatus* (Say)*,* Sebring strain, were reared and maintained in a laboratory under controlled temperature (27 °C) and relative humidity (70%) with a photoperiod of 14:10 (L:D) h. Adults were housed in 31 cm^3^ collapsible cages and provided a 10% sucrose solution ad libitum from cotton dental wicks. Cages were draped with damp cloth covered with plastic bags to maintain humidity. Mosquitoes were provided defibrinated blood, chicken for *Cx. quinquefasciatus* and sheep for *Ae. aegypti*, once a week using an artificial feeding system (Hemotek^®^ Ltd, England) with Parafilm^®^ (Bemis Company, Oshkosh, WI). Three to 5-day-old female mosquitoes were used in assays. The sucrose solution was removed 18 h prior to the commencement of the tests.

For USDA laboratory assays, Diptera from established colonies were used. Colonies of *Cx. tarsalis* and *Cx. quinquefasciatus* were established from material provided by UC Davis^47^. Larvae were fed a 1:1 ratio by volume or ground fish flakes to ground cat food and reared at 26 °C. Adults were maintained at 26 °C, 70% relative humidity, and a photoperiod of 13:11 (L:D) h. The colony of *Culicoides sonorensis* (Wirth and Jones) established by the USDA Arthropod-Borne Animal Disease Research Unit (ABADRU) from material collected in Owyhee County, ID in 1973 was used for biting midge assays. Midges were reared using a modified Hunt’s method^48^. Briefly, larvae were reared on a bacterial inoculum at 28 °C. Adults were maintained at 26 °C, 70% relative humidity, and a photoperiod of 13:11 (L:D) h. *Musca domestica* L. came from the ABADRU colony, a blended line established from Georgia and Kansas. Larvae were reared in wheat bran supplemented with commercial calf feed. Larvae and adults were maintained at 28 °C, 70% relative humidity, and a photoperiod of 13:11. For each species, pupae were transferred to emergence cages and given ad libitum access to 10% sucrose water solution. For mosquitoes and house flies 3 to 5-day-old adults were used. Only females were used for mosquitoes, and both sexes were used for house flies. For biting midges, 2 to 3-day-old females were used. Flies were starved of sucrose water 18 h prior to feeding assays.

### Sugar alcohol preparation

Sugar alcohols were obtained from the following sources: erythritol (100% food grade, Pyure Brands LLC, Naples FL, USA), sorbitol (100% food grade, Bulk Supplements, Henderson, NV, USA), xylitol (100% food grade, Now Foods, Bloomingdale, IL, USA), and d-mannitol (CAS No. 69-65-8, 100% purity, HiMedia Laboratories, Mumbai, India). Assay solutions were prepared by adding each of the four sugar alcohols to a 10% (w/v) sucrose solution (white granulated sugar (Great Value, Walmart-Store Inc, Bentonville, AR, USA) to water). Sucrose solution is used to distinguish mortality due to toxicological effects of sugar alcohols from starvation, and was also found to facilitate the insecticidal properties of sugar alcohols^27,33,34^. To the sucrose solution, the various sugar alcohols were added to make 10%, 20%, and 30% (w/v) solutions. The sugar alcohols dissolved into the sucrose mixture in all solutions by gentle mixing excepting 20% and 30% d-mannitol, for which the glass bottles holding the solution were heated to approximately 95 °C and swirled intermittently until the contents were clear, approximately 5 min. To all of the sugar alcohol solutions, 1% (v/v) of red, green, or blue food coloring (McCormick & Company, Inc, Hunt Valley, MD, USA) was added to confirm consumption of the solutions (Fig. 1).

### Sugar alcohol feeding and survivorship assays

**Figure 4:**
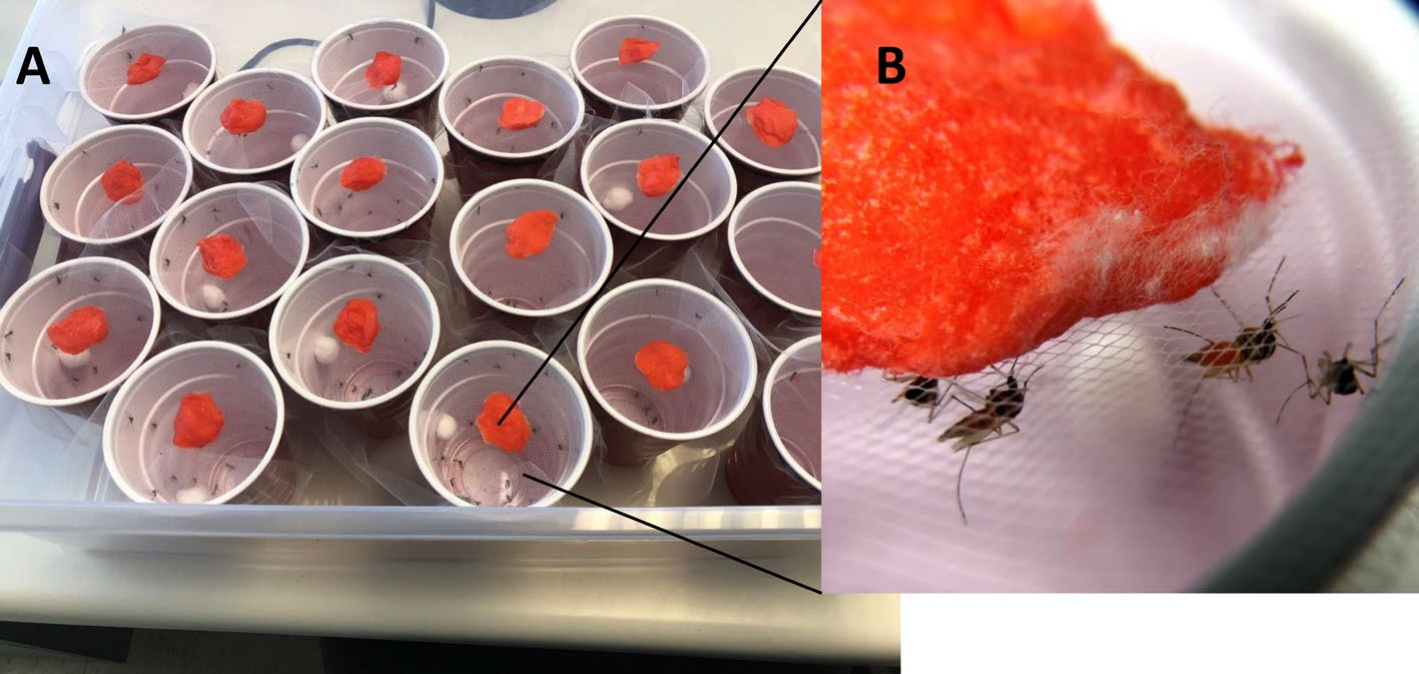
Experimental bioassays. (**A**) Multiple cups with feeding solution with added red food dye. (**B**) Adult female mosquitoes feeding on the mixture of sugar alcohols and sucrose solution.

Assays were conducted using 350 ml disposable plastic drinking cups covered with tulle mesh secured by a rubber band. For each species, 10–30 insects were transferred to each container (number varied due to availability and species size). A cotton ball saturated, yet not dripping with a test solution was placed on top of the tulle cover. Three replicate cups were made for each sugar alcohol solution and species combination. The cups were placed in 30 L plastic storage totes, which were then held in an insectary at 27 °C (Fig. 4). Cotton balls with the test solutions were replaced every 24 h with fresh solution. Mortality was recorded at 24 h intervals by tapping the sides and bottom of the cup to check for movement. Moribund mosquitoes (falling or lying on their backs without getting up or having erratic, uncontrollable flight) were considered dead. Two control assays of 10% (w/v) sucrose with 1% (v/v) food coloring and water with 1% v/v food coloring also were performed.

### Statistical analysis

Mortality rates were compared between the treatments and the controls using mixed effects model generated by package lme4 v. 1.1-10^49^. The full generalized linear mixed model contained three-way interaction and the main effects of species, treatment, and time as fixed effects. Random effects included time and replicates nested within each trial to account for the hierarchical experimental structure.

$${\text{Mortality }}\sim {\text{ Species }} \times {\text{ Treatment }} \times {\text{ Time }} + \, \left( {{\text{time}}|{\text{experiment}}} \right) \, + \, \left( {{1}|{\text{experiment}}/{\text{replica}}} \right)$$

The full model contained random intercept and random slope to account for potential differences in mortalities among different experiments and locations. The proportion of dead vs. live insects observed daily was used as response variable in the model with binomial distribution. To check the model’s assumptions, residual plots were visually inspected for obvious deviations from homoscedasticity or normality. Post hoc tests were performed by planned contrasts with adjusted P-values by Tukey’s range test using package emmeans v.1.4.7.

Survival analyses were conducted using the Kaplan–Meier analysis and Cox proportional-hazards modeling performed in R statistical software using the survival (version 3.1–12)^50^ and survminer (version 0.4.9)^51^ packages. In the survival model, time and occurrence of death were included as response variables, while the treatment (sugar alcohols, water, or sucrose solution) were the explanatory variables. Individuals who did not die by the end of the experiment were censored (0 = death event did not occur; 1 = death event occurred). Subsequent pairwise comparisons using log-ranks test were corrected using Benjamini–Hochberg corrections to reduce Type I error rate. All graphics were generated in R using the ggsurvplot function within the survminer package.

## Supplementary Information

Supplementary Table 1.
