# Supplementary material for: Laboratory evaluation of sugar alcohols for control of mosquitoes and other medically important flies
Source: Sci Rep. 2022 Aug 12;12:13763. doi: 10.1038/s41598-022-15825-z (PMC9374714; doi:10.1038/s41598-022-15825-z)
Supplement: Supplementary file 1 — Supplementary Table 1. [file 41598_2022_15825_MOESM1_ESM.docx]

**Laboratory evaluation of sugar alcohols for control of mosquitoes and other medically important flies**

Ilia Rochlin, Greg White, Nadja Reissen, Dustin Swanson, Lee Cohnstaedt, Madeline Chura, Kristen Healy, and Ary Faraji

**Supplementary Table 1. Percent mortality by species, treatment, and time.** AEG - *Ae. aegypti*, CU- *Cu. sonorensis*, DOR – *Ae. dorsalis*, MU – *Mu. domestica*, QNQ – *Cx. quinquefasciatus*, TAR – *Cx. tarsalis*.

| Species | Time | Treatment | Concentration | Mean %  mortality | SE |
| --- | --- | --- | --- | --- | --- |
| AEG | 1 | sucrose | NA | 1.3 | 1.3 |
| AEG | 1 | water | NA | 2.0 | 2.0 |
| AEG | 1 | sorbitol | 10 | 0.0 | 0.0 |
| AEG | 1 | sorbitol | 20 | 0.0 | 0.0 |
| AEG | 1 | sorbitol | 30 | 1.3 | 1.3 |
| AEG | 1 | xylitol | 10 | 0.0 | 0.0 |
| AEG | 1 | xylitol | 20 | 2.0 | 1.0 |
| AEG | 1 | xylitol | 30 | 0.7 | 0.7 |
| AEG | 1 | erythritol | 10 | 2.7 | 1.6 |
| AEG | 1 | erythritol | 20 | 2.2 | 0.6 |
| AEG | 1 | erythritol | 30 | 21.5 | 0.5 |
| AEG | 2 | sucrose | NA | 2.6 | 2.6 |
| AEG | 2 | water | NA | 7.4 | 5.5 |
| AEG | 2 | sorbitol | 10 | 1.5 | 1.5 |
| AEG | 2 | sorbitol | 20 | 0.0 | 0.0 |
| AEG | 2 | sorbitol | 30 | 14.4 | 2.4 |
| AEG | 2 | xylitol | 10 | 1.2 | 1.2 |
| AEG | 2 | xylitol | 20 | 7.5 | 2.7 |
| AEG | 2 | xylitol | 30 | 8.0 | 6.7 |
| AEG | 2 | erythritol | 10 | 8.2 | 1.5 |
| AEG | 2 | erythritol | 20 | 30.9 | 0.9 |
| AEG | 2 | erythritol | 30 | 46.0 | 1.1 |
| AEG | 3 | sucrose | NA | 3.8 | 3.8 |
| AEG | 3 | water | NA | 18.6 | 6.3 |
| AEG | 3 | sorbitol | 10 | 4.5 | 2.6 |
| AEG | 3 | sorbitol | 20 | 0.0 | 0.0 |
| AEG | 3 | sorbitol | 30 | 46.9 | 3.9 |
| AEG | 3 | xylitol | 10 | 6.0 | 4.3 |
| AEG | 3 | xylitol | 20 | 32.5 | 2.6 |
| AEG | 3 | xylitol | 30 | 45.9 | 6.0 |
| AEG | 3 | erythritol | 10 | 42.1 | 0.7 |
| AEG | 3 | erythritol | 20 | 65.4 | 0.4 |
| AEG | 3 | erythritol | 30 | 73.0 | 0.5 |
| CU | 1 | sucrose | NA | 0.0 | 0.0 |
| CU | 1 | water | NA | 0.7 | 0.7 |
| CU | 1 | d-mannitol | 10 | 2.5 | 2.5 |
| CU | 1 | d-mannitol | 20 | 6.5 | 1.2 |
| CU | 1 | d-mannitol | 30 | 2.7 | 1.4 |
| CU | 1 | sorbitol | 10 | 0.0 | 0.0 |
| CU | 1 | sorbitol | 20 | 2.4 | 2.4 |
| CU | 1 | sorbitol | 30 | 0.0 | 0.0 |
| CU | 1 | xylitol | 10 | 1.3 | 1.3 |
| CU | 1 | xylitol | 20 | 3.6 | 1.9 |
| CU | 1 | xylitol | 30 | 1.0 | 1.0 |
| CU | 1 | erythritol | 10 | 0.0 | 0.0 |
| CU | 1 | erythritol | 20 | 5.1 | 1.6 |
| CU | 1 | erythritol | 30 | 5.6 | 1.3 |
| CU | 2 | sucrose | NA | 2.7 | 1.4 |
| CU | 2 | water | NA | 6.3 | 1.1 |
| CU | 2 | d-mannitol | 10 | 6.3 | 3.3 |
| CU | 2 | d-mannitol | 20 | 10.4 | 1.2 |
| CU | 2 | d-mannitol | 30 | 5.3 | 1.2 |
| CU | 2 | sorbitol | 10 | 1.4 | 1.4 |
| CU | 2 | sorbitol | 20 | 6.2 | 4.2 |
| CU | 2 | sorbitol | 30 | 1.4 | 1.4 |
| CU | 2 | xylitol | 10 | 4.4 | 2.3 |
| CU | 2 | xylitol | 20 | 7.2 | 3.9 |
| CU | 2 | xylitol | 30 | 10.6 | 3.2 |
| CU | 2 | erythritol | 10 | 1.4 | 1.4 |
| CU | 2 | erythritol | 20 | 11.8 | 2.2 |
| CU | 2 | erythritol | 30 | 21.7 | 1.3 |
| CU | 3 | sucrose | NA | 6.7 | 3.5 |
| CU | 3 | water | NA | 25.3 | 2.0 |
| CU | 3 | d-mannitol | 10 | 11.3 | 5.7 |
| CU | 3 | d-mannitol | 20 | 15.5 | 1.9 |
| CU | 3 | d-mannitol | 30 | 12.0 | 1.9 |
| CU | 3 | sorbitol | 10 | 2.9 | 2.9 |
| CU | 3 | sorbitol | 20 | 11.6 | 5.1 |
| CU | 3 | sorbitol | 30 | 2.9 | 2.9 |
| CU | 3 | xylitol | 10 | 7.5 | 3.8 |
| CU | 3 | xylitol | 20 | 11.9 | 6.7 |
| CU | 3 | xylitol | 30 | 23.8 | 8.4 |
| CU | 3 | erythritol | 10 | 5.5 | 2.7 |
| CU | 3 | erythritol | 20 | 19.8 | 3.8 |
| CU | 3 | erythritol | 30 | 41.3 | 3.2 |
| CU | 4 | sucrose | NA | 12.0 | 6.7 |
| CU | 4 | water | NA | 57.7 | 1.1 |
| CU | 4 | d-mannitol | 10 | 16.3 | 8.2 |
| CU | 4 | d-mannitol | 20 | 22.0 | 2.0 |
| CU | 4 | d-mannitol | 30 | 18.7 | 3.0 |
| CU | 4 | sorbitol | 10 | 4.3 | 4.3 |
| CU | 4 | sorbitol | 20 | 16.9 | 5.9 |
| CU | 4 | sorbitol | 30 | 4.3 | 4.3 |
| CU | 4 | xylitol | 10 | 13.7 | 6.8 |
| CU | 4 | xylitol | 20 | 16.7 | 9.6 |
| CU | 4 | xylitol | 30 | 37.6 | 14.2 |
| CU | 4 | erythritol | 10 | 9.5 | 4.8 |
| CU | 4 | erythritol | 20 | 27.8 | 5.8 |
| CU | 4 | erythritol | 30 | 62.5 | 6.3 |
| DOR | 1 | sucrose | NA | 7.3 | 1.6 |
| DOR | 1 | water | NA | 9.1 | 1.2 |
| DOR | 1 | d-mannitol | 10 | 13.1 | 1.5 |
| DOR | 1 | d-mannitol | 20 | 9.4 | 2.3 |
| DOR | 1 | d-mannitol | 30 | 9.6 | 1.0 |
| DOR | 1 | sorbitol | 10 | 14.2 | 1.8 |
| DOR | 1 | sorbitol | 20 | 14.2 | 1.5 |
| DOR | 1 | sorbitol | 30 | 11.1 | 1.2 |
| DOR | 1 | xylitol | 10 | 9.0 | 1.4 |
| DOR | 1 | xylitol | 20 | 11.1 | 1.2 |
| DOR | 1 | xylitol | 30 | 11.6 | 1.6 |
| DOR | 1 | erythritol | 10 | 9.7 | 1.1 |
| DOR | 1 | erythritol | 20 | 11.4 | 1.3 |
| DOR | 1 | erythritol | 30 | 16.7 | 0.9 |
| DOR | 2 | sucrose | NA | 16.0 | 3.1 |
| DOR | 2 | water | NA | 23.5 | 2.9 |
| DOR | 2 | d-mannitol | 10 | 28.4 | 2.1 |
| DOR | 2 | d-mannitol | 20 | 22.0 | 3.8 |
| DOR | 2 | d-mannitol | 30 | 22.4 | 1.9 |
| DOR | 2 | sorbitol | 10 | 29.7 | 3.1 |
| DOR | 2 | sorbitol | 20 | 29.6 | 2.8 |
| DOR | 2 | sorbitol | 30 | 22.1 | 2.5 |
| DOR | 2 | xylitol | 10 | 20.8 | 2.7 |
| DOR | 2 | xylitol | 20 | 27.4 | 1.8 |
| DOR | 2 | xylitol | 30 | 27.9 | 3.2 |
| DOR | 2 | erythritol | 10 | 26.0 | 2.6 |
| DOR | 2 | erythritol | 20 | 32.2 | 2.4 |
| DOR | 2 | erythritol | 30 | 42.4 | 0.9 |
| DOR | 3 | sucrose | NA | 28.9 | 3.9 |
| DOR | 3 | water | NA | 44.4 | 5.5 |
| DOR | 3 | d-mannitol | 10 | 45.4 | 2.0 |
| DOR | 3 | d-mannitol | 20 | 37.3 | 5.5 |
| DOR | 3 | d-mannitol | 30 | 39.5 | 2.5 |
| DOR | 3 | sorbitol | 10 | 48.8 | 3.4 |
| DOR | 3 | sorbitol | 20 | 48.3 | 3.6 |
| DOR | 3 | sorbitol | 30 | 36.4 | 2.8 |
| DOR | 3 | xylitol | 10 | 34.6 | 3.5 |
| DOR | 3 | xylitol | 20 | 44.4 | 2.7 |
| DOR | 3 | xylitol | 30 | 47.1 | 5.5 |
| DOR | 3 | erythritol | 10 | 52.7 | 3.0 |
| DOR | 3 | erythritol | 20 | 62.7 | 1.6 |
| DOR | 3 | erythritol | 30 | 71.1 | 0.4 |
| MU | 1 | sucrose | NA | 0.0 | 0.0 |
| MU | 1 | water | NA | 18.0 | 1.0 |
| MU | 1 | d-mannitol | 10 | 0.0 | 0.0 |
| MU | 1 | d-mannitol | 20 | 3.4 | 3.4 |
| MU | 1 | d-mannitol | 30 | 2.0 | 2.0 |
| MU | 1 | sorbitol | 10 | 0.0 | 0.0 |
| MU | 1 | sorbitol | 20 | 1.6 | 1.6 |
| MU | 1 | sorbitol | 30 | 6.7 | 3.3 |
| MU | 1 | xylitol | 10 | 0.0 | 0.0 |
| MU | 1 | xylitol | 20 | 3.0 | 3.0 |
| MU | 1 | xylitol | 30 | 8.5 | 8.5 |
| MU | 1 | erythritol | 10 | 0.0 | 0.0 |
| MU | 1 | erythritol | 20 | 3.4 | 1.8 |
| MU | 1 | erythritol | 30 | 7.1 | 3.6 |
| MU | 2 | sucrose | NA | 0.0 | 0.0 |
| MU | 2 | water | NA | 37.5 | 1.0 |
| MU | 2 | d-mannitol | 10 | 0.0 | 0.0 |
| MU | 2 | d-mannitol | 20 | 6.8 | 6.8 |
| MU | 2 | d-mannitol | 30 | 3.9 | 3.9 |
| MU | 2 | sorbitol | 10 | 1.4 | 1.4 |
| MU | 2 | sorbitol | 20 | 3.9 | 3.9 |
| MU | 2 | sorbitol | 30 | 11.1 | 5.6 |
| MU | 2 | xylitol | 10 | 0.0 | 0.0 |
| MU | 2 | xylitol | 20 | 7.6 | 7.6 |
| MU | 2 | xylitol | 30 | 16.9 | 16.9 |
| MU | 2 | erythritol | 10 | 0.0 | 0.0 |
| MU | 2 | erythritol | 20 | 5.8 | 2.9 |
| MU | 2 | erythritol | 30 | 22.5 | 5.5 |
| MU | 3 | sucrose | NA | 0.0 | 0.0 |
| MU | 3 | water | NA | 58.3 | 0.7 |
| MU | 3 | d-mannitol | 10 | 0.0 | 0.0 |
| MU | 3 | d-mannitol | 20 | 11.1 | 11.1 |
| MU | 3 | d-mannitol | 30 | 11.5 | 6.8 |
| MU | 3 | sorbitol | 10 | 2.9 | 2.9 |
| MU | 3 | sorbitol | 20 | 7.0 | 7.0 |
| MU | 3 | sorbitol | 30 | 19.0 | 4.4 |
| MU | 3 | xylitol | 10 | 1.5 | 1.5 |
| MU | 3 | xylitol | 20 | 14.5 | 11.1 |
| MU | 3 | xylitol | 30 | 28.2 | 22.6 |
| MU | 3 | erythritol | 10 | 0.0 | 0.0 |
| MU | 3 | erythritol | 20 | 12.3 | 3.4 |
| MU | 3 | erythritol | 30 | 39.4 | 9.1 |
| MU | 4 | sucrose | NA | 0.0 | 0.0 |
| MU | 4 | water | NA | 79.2 | 0.3 |
| MU | 4 | d-mannitol | 10 | 1.6 | 1.6 |
| MU | 4 | d-mannitol | 20 | 26.5 | 12.7 |
| MU | 4 | d-mannitol | 30 | 22.4 | 12.0 |
| MU | 4 | sorbitol | 10 | 7.4 | 3.9 |
| MU | 4 | sorbitol | 20 | 17.8 | 17.8 |
| MU | 4 | sorbitol | 30 | 32.5 | 1.5 |
| MU | 4 | xylitol | 10 | 3.0 | 3.0 |
| MU | 4 | xylitol | 20 | 30.7 | 13.2 |
| MU | 4 | xylitol | 30 | 56.8 | 12.4 |
| MU | 4 | erythritol | 10 | 0.0 | 0.0 |
| MU | 4 | erythritol | 20 | 31.2 | 7.3 |
| MU | 4 | erythritol | 30 | 59.9 | 10.3 |
| QNQ | 1 | sucrose | NA | 1.5 | 0.7 |
| QNQ | 1 | water | NA | 1.6 | 0.6 |
| QNQ | 1 | d-mannitol | 10 | 2.3 | 2.3 |
| QNQ | 1 | d-mannitol | 20 | 2.5 | 1.3 |
| QNQ | 1 | d-mannitol | 30 | 4.3 | 2.2 |
| QNQ | 1 | sorbitol | 10 | 0.0 | 0.0 |
| QNQ | 1 | sorbitol | 20 | 0.0 | 0.0 |
| QNQ | 1 | sorbitol | 30 | 0.0 | 0.0 |
| QNQ | 1 | xylitol | 10 | 4.6 | 1.9 |
| QNQ | 1 | xylitol | 20 | 3.6 | 1.9 |
| QNQ | 1 | xylitol | 30 | 5.3 | 2.1 |
| QNQ | 1 | erythritol | 10 | 3.6 | 1.5 |
| QNQ | 1 | erythritol | 20 | 5.1 | 1.0 |
| QNQ | 1 | erythritol | 30 | 14.6 | 4.0 |
| QNQ | 2 | sucrose | NA | 3.4 | 1.3 |
| QNQ | 2 | water | NA | 8.4 | 1.6 |
| QNQ | 2 | d-mannitol | 10 | 4.6 | 4.6 |
| QNQ | 2 | d-mannitol | 20 | 6.2 | 3.2 |
| QNQ | 2 | d-mannitol | 30 | 11.9 | 6.1 |
| QNQ | 2 | sorbitol | 10 | 1.3 | 0.9 |
| QNQ | 2 | sorbitol | 20 | 2.3 | 1.7 |
| QNQ | 2 | sorbitol | 30 | 2.8 | 1.5 |
| QNQ | 2 | xylitol | 10 | 9.3 | 3.8 |
| QNQ | 2 | xylitol | 20 | 8.0 | 3.6 |
| QNQ | 2 | xylitol | 30 | 16.9 | 5.0 |
| QNQ | 2 | erythritol | 10 | 13.1 | 2.8 |
| QNQ | 2 | erythritol | 20 | 27.1 | 4.2 |
| QNQ | 2 | erythritol | 30 | 35.8 | 5.8 |
| QNQ | 3 | sucrose | NA | 6.6 | 2.1 |
| QNQ | 3 | water | NA | 26.8 | 3.2 |
| QNQ | 3 | d-mannitol | 10 | 8.4 | 6.3 |
| QNQ | 3 | d-mannitol | 20 | 11.1 | 6.0 |
| QNQ | 3 | d-mannitol | 30 | 19.4 | 10.0 |
| QNQ | 3 | sorbitol | 10 | 4.2 | 1.5 |
| QNQ | 3 | sorbitol | 20 | 6.3 | 3.6 |
| QNQ | 3 | sorbitol | 30 | 9.7 | 3.4 |
| QNQ | 3 | xylitol | 10 | 15.0 | 5.9 |
| QNQ | 3 | xylitol | 20 | 13.6 | 5.4 |
| QNQ | 3 | xylitol | 30 | 30.0 | 8.5 |
| QNQ | 3 | erythritol | 10 | 39.4 | 6.0 |
| QNQ | 3 | erythritol | 20 | 50.9 | 7.8 |
| QNQ | 3 | erythritol | 30 | 58.8 | 7.1 |
| QNQ | 4 | sucrose | NA | 6.7 | 6.7 |
| QNQ | 4 | water | NA | 32.8 | 16.8 |
| QNQ | 4 | d-mannitol | 10 | 13.4 | 9.2 |
| QNQ | 4 | d-mannitol | 20 | 15.9 | 9.0 |
| QNQ | 4 | d-mannitol | 30 | 29.2 | 6.4 |
| QNQ | 4 | sorbitol | 10 | 7.4 | 3.9 |
| QNQ | 4 | sorbitol | 20 | 10.3 | 10.3 |
| QNQ | 4 | sorbitol | 30 | 8.6 | 4.8 |
| QNQ | 4 | xylitol | 10 | 8.6 | 8.6 |
| QNQ | 4 | xylitol | 20 | 4.3 | 4.3 |
| QNQ | 4 | xylitol | 30 | 18.5 | 3.8 |
| QNQ | 4 | erythritol | 10 | 39.3 | 5.6 |
| QNQ | 4 | erythritol | 20 | 51.7 | 5.0 |
| QNQ | 4 | erythritol | 30 | 64.9 | 0.7 |
| TAR | 1 | sucrose | NA | 5.6 | 1.4 |
| TAR | 1 | water | NA | 3.3 | 1.0 |
| TAR | 1 | d-mannitol | 10 | 6.7 | 1.1 |
| TAR | 1 | d-mannitol | 20 | 6.2 | 1.7 |
| TAR | 1 | d-mannitol | 30 | 4.5 | 1.2 |
| TAR | 1 | sorbitol | 10 | 3.8 | 1.1 |
| TAR | 1 | sorbitol | 20 | 5.4 | 1.9 |
| TAR | 1 | sorbitol | 30 | 6.2 | 2.0 |
| TAR | 1 | xylitol | 10 | 3.2 | 1.1 |
| TAR | 1 | xylitol | 20 | 5.2 | 1.5 |
| TAR | 1 | xylitol | 30 | 7.4 | 1.4 |
| TAR | 1 | erythritol | 10 | 6.5 | 1.9 |
| TAR | 1 | erythritol | 20 | 10.1 | 2.0 |
| TAR | 1 | erythritol | 30 | 14.4 | 2.3 |
| TAR | 2 | sucrose | NA | 12.1 | 2.8 |
| TAR | 2 | water | NA | 11.1 | 3.4 |
| TAR | 2 | d-mannitol | 10 | 15.8 | 2.2 |
| TAR | 2 | d-mannitol | 20 | 15.2 | 3.9 |
| TAR | 2 | d-mannitol | 30 | 11.3 | 2.6 |
| TAR | 2 | sorbitol | 10 | 8.9 | 2.2 |
| TAR | 2 | sorbitol | 20 | 11.1 | 3.4 |
| TAR | 2 | sorbitol | 30 | 12.5 | 3.8 |
| TAR | 2 | xylitol | 10 | 7.6 | 2.1 |
| TAR | 2 | xylitol | 20 | 13.0 | 3.2 |
| TAR | 2 | xylitol | 30 | 21.3 | 3.4 |
| TAR | 2 | erythritol | 10 | 17.7 | 3.9 |
| TAR | 2 | erythritol | 20 | 28.3 | 4.4 |
| TAR | 2 | erythritol | 30 | 37.5 | 3.3 |
| TAR | 3 | sucrose | NA | 21.2 | 4.5 |
| TAR | 3 | water | NA | 32.5 | 5.1 |
| TAR | 3 | d-mannitol | 10 | 27.1 | 3.4 |
| TAR | 3 | d-mannitol | 20 | 26.7 | 5.6 |
| TAR | 3 | d-mannitol | 30 | 19.2 | 3.9 |
| TAR | 3 | sorbitol | 10 | 15.2 | 3.7 |
| TAR | 3 | sorbitol | 20 | 17.7 | 5.2 |
| TAR | 3 | sorbitol | 30 | 19.4 | 5.4 |
| TAR | 3 | xylitol | 10 | 14.0 | 3.2 |
| TAR | 3 | xylitol | 20 | 22.4 | 4.8 |
| TAR | 3 | xylitol | 30 | 35.9 | 5.0 |
| TAR | 3 | erythritol | 10 | 34.5 | 6.4 |
| TAR | 3 | erythritol | 20 | 52.4 | 6.5 |
| TAR | 3 | erythritol | 30 | 63.9 | 4.0 |
| TAR | 4 | sucrose | NA | 19.1 | 10.9 |
| TAR | 4 | water | NA | 48.1 | 12.8 |
| TAR | 4 | d-mannitol | 10 | 16.5 | 3.5 |
| TAR | 4 | d-mannitol | 20 | 3.0 | 3.0 |
| TAR | 4 | d-mannitol | 30 | 5.6 | 5.6 |
| TAR | 4 | sorbitol | 10 | 16.3 | 8.2 |
| TAR | 4 | sorbitol | 20 | 0.0 | 0.0 |
| TAR | 4 | sorbitol | 30 | 4.3 | 4.3 |
| TAR | 4 | xylitol | 10 | 9.0 | 5.6 |
| TAR | 4 | xylitol | 20 | 4.3 | 4.3 |
| TAR | 4 | xylitol | 30 | 31.1 | 13.5 |
| TAR | 4 | erythritol | 10 | 15.5 | 7.0 |
| TAR | 4 | erythritol | 20 | 33.6 | 16.9 |
| TAR | 4 | erythritol | 30 | 65.2 | 12.0 |
